# Unveiling the microevolution of antimicrobial resistance in selected *Pseudomonas aeruginosa* isolates from Egyptian healthcare settings: A genomic approach

**DOI:** 10.1038/s41598-024-65178-y

**Published:** 2024-07-05

**Authors:** Salma Salem, Nehal Adel Abdelsalam, Ahmed H. Shata, Shaimaa F. Mouftah, José F. Cobo-Díaz, Dina Osama, Reham Atteya, Mohamed Elhadidy

**Affiliations:** 1 Center for Genomics, Helmy Institute for Medical Sciences, Zewail City of Science and Technology, Giza, Egypt; 2 Biomedical Sciences Program, University of Science and Technology, Zewail City of Science and Technology, Giza, Egypt; 3 Department of Microbiology and Immunology, Faculty of Pharmacy, Cairo University, Cairo, Egypt; 4 Department of Food Hygiene and Technology, Institute of Food Science and Technology, Universidad de León, León, Spain; 5 Department of Microbiology and Immunology, Faculty of Pharmacy, October University for Modern Sciences and Arts (MSA), Cairo, Egypt; 6 Department of Bacteriology, Mycology and Immunology, Faculty of Veterinary Medicine, Mansoura University, Mansoura, Egypt

**Keywords:** *Pseudomonas aeruginos*a, Sequence typing, Antimicrobial resistance, Biofilm formation, Virulome, Horizontal gene transfer, Evolution, Microbiology

## Abstract

The incidence of *Pseudomonas aeruginosa* infections in healthcare environments, particularly in low-and middle-income countries, is on the rise. The purpose of this study was to provide comprehensive genomic insights into thirteen *P. aeruginosa* isolates obtained from Egyptian healthcare settings. Phenotypic analysis of the antimicrobial resistance profile and biofilm formation were performed using minimum inhibitory concentration and microtiter plate assay, respectively. Whole genome sequencing was employed to identify sequence typing, resistome, virulome, and mobile genetic elements. Our findings indicate that 92.3% of the isolates were classified as extensively drug-resistant, with 53.85% of these demonstrating strong biofilm production capabilities. The predominant clone observed in the study was ST773, followed by ST235, both of which were associated with the O11 serotype. Core genome multi-locus sequence typing comparison of these clones with global isolates suggested their potential global expansion and adaptation. A significant portion of the isolates harbored Col plasmids and various MGEs, all of which were linked to antimicrobial resistance genes. Single nucleotide polymorphisms in different genes were associated with the development of antimicrobial resistance in these isolates. In conclusion, this pilot study underscores the prevalence of extensively drug-resistant *P. aeruginosa* isolates and emphasizes the role of horizontal gene transfer facilitated by a diverse array of mobile genetic elements within various clones. Furthermore, specific insertion sequences and mutations were found to be associated with antibiotic resistance.

## Introduction

*Pseudomonas aeruginosa* (*P. aeruginosa*) represents a ubiquitous gram-negative bacterium in hospital-acquired infections, surgical and transplantation-related infections, as well as instances of ventilator-associated pneumonia. Furthermore, this bacterial pathogen poses a substantial risk to immunocompromised patients with conditions such as cystic fibrosis, burns, and sepsis^1–5^. The ability of *P. aeruginosa* to thrive in harsh environments through biofilm formation enhances its pathogenicity^6^. As one of the multidrug-resistant ESKAPE pathogens, alongside *Enterococcus faecium*, *Staphylococcus aureus*, *Klebsiella pneumoniae*, *Acinetobacter baumannii*, and *Enterobacter* species, *P. aeruginosa* poses a challenge in the healthcare settings^7^.

*P. aeruginosa* expresses various virulence factors, including secretion systems such as type II, type III, and type VI secretion systems. Type III secretion system (T3SS) plays a significant role in the bacterial pathogenicity by directly injecting toxins into eukaryotic cells. This process facilitates the invasion of host cells and establishment of infection^8^. Moreover, the type II secretion system (T2SS) pathway is responsible for the secretion of toxins like exotoxin A, LasA protease, and LasB protease, which are encoded by *toxA, lasA*, and *lasB*, respectively. Interestingly, this pathway relies on the activity of the macromolecular complex Xcp secretion^9^. Moreover, it encodes three types of type VI secretion systems (T6SS), designated as H1, H2, and H3. These systems comprise tightly regulated gene clusters containing 13 conserved genes, which serve as core components. The hemolysin‐coregulated protein (Hcp) protein is considered a hallmark of T6SS and can be used as a biomarker to indicate T6SS activation. The H1-T6SS specifically secretes the effector protein Hcp1, and this secretion process is facilitated by the ClpV1 protein^10,11^. Additionally, this bacterium relies on two phospholipase D enzymes, PldA and PldB, for host-cell invasion. These enzymes are delivered into target cells via the H2-T6SS or H3-T6SS, respectively. Notably, T6SS has the ability target both prokaryotic and eukaryotic cells^10^. In addition to these virulence factors, *P. aeruginosa* secretes toxins such as exotoxin A, exoenzymes, phospholipases C, and pyocyanin, which are harmful to host cells and contribute to tissue damage and inflammation during infection^12,13^.

Managing multiple-drug resistant (MDR) P*. aeruginosa* has become increasingly challenging in both nosocomial and community-acquired settings due to various mechanisms of antibiotic resistance (AMR)^14,15^. The mechanisms of intrinsic AMR include reduced outer membrane permeability, efflux systems actively pump drugs out of the bacterial cells, and the production of enzymes that inhibit the action of antibiotics. Secondly, the microevolution of antibiotic resistance can occur through the accumulation of mutational changes or by acquisition of resistance genes from other bacteria through horizontal gene transfer. The combination of intrinsic and acquired resistance mechanisms contributes to the emergence of MDR strains. Lastly, adaptive resistance enhances the bacterial ability to resist antibiotics through biofilm formation or generation of persister cell^16–19^.

The mechanisms of antibiotic tolerance associated with biofilms depend on physical, physiological, and genetic determinants. The structure of the biofilms acts as a physical barrier and prevents the antibiotics from penetrating deeper layers of the biofilm. The extracellular matrix serves as a protective shield and prevents the antibiotics from reaching to the bacterial cells. Therefore, physical barriers reduce the effectiveness of antibiotics against biofilm-associated bacteria. Moreover, recent studies have established a correlation between horizontally acquired resistance and AMR genotype^20–22^.

This study aimed to screen the antimicrobial susceptibility pattern and biofilm formation of thirteen non-clonal *P. aeruginosa* isolates obtained from healthcare facilities in Egypt. Additionally, we investigated the clonal distribution, presence of virulence factors, and various genetic determinants contributing to AMR, encompassing the acquisition of antibiotic resistance genes and genetic mutations.

## Results

### Genomic features

The total genome assembly size of the *P. aeruginosa* isolates was 6.78 ± 0.5 Mbp with 66% GC content. The number of contigs ranged from 157 to 886. The N50 was between 12,258 and 67,663 bp, whereas L50 ranged from 30 to 142 (Table S1). The core-genome multi-locus sequence typing (cgMLST) profiles of *P. aeruginosa* isolates were based on 3865 out of a total of 5573 genes. The pangenome of the isolates consisted of 4479 core genes, 3502 shell genes, and 2044 cloud genes.

### Antimicrobial resistance and carbapenemase production

All the screened isolates showed high resistance to ticarcillin, ticarcillin-clavulanic acid, and aztreonam. Most of the isolates (12/13; 92.3%) exhibited notable resistance to piperacillin, piperacillin-tazobactam, ceftazidime, cefepime, amikacin, gentamicin, tobramycin, ciprofloxacin, imipenem, and meropenem. On the other hand, all isolates were susceptible to colistin. Most of the isolates (12/13; 92.3%) were classified as extensively drug-resistant (XDR). Using modified carbapenem inactivation method (mCIM) assay, (4/13; 30.77%) of screened isolates were confirmed to produce carbapenemase (Table S2).

### MLST and serotyping

**Figure 1:**
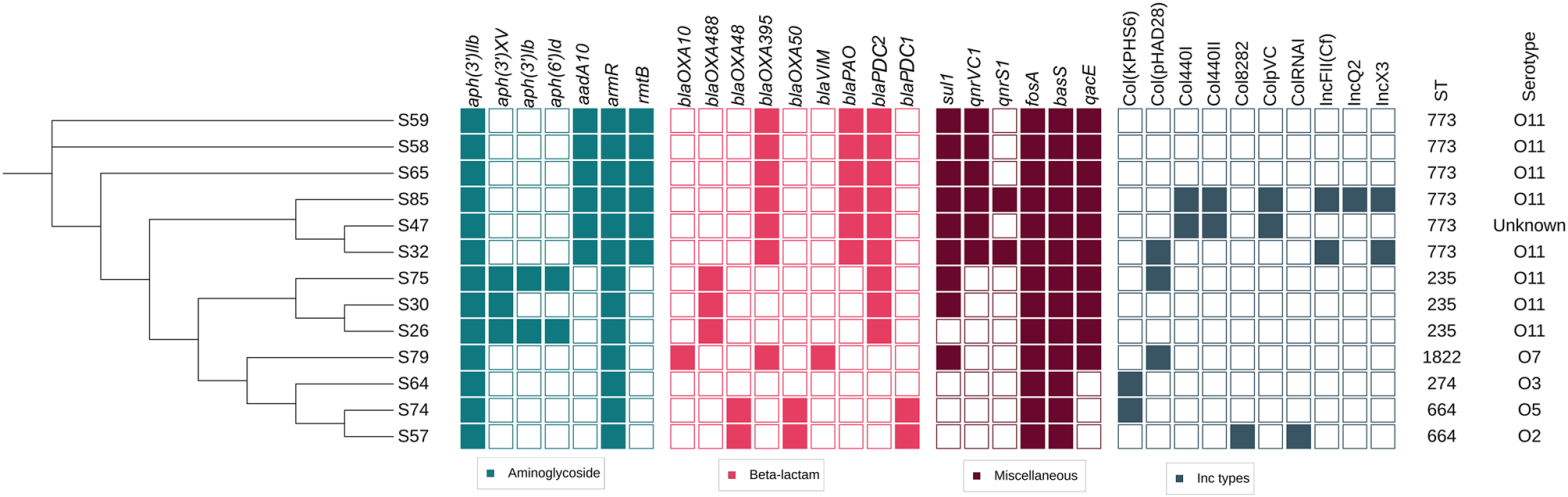
Phylogenetic tree illustrating the distribution of antimicrobial resistance genes, serotypes and plasmids replicon among different STs. Inc types found within all the isolates are represented. Cerulean, pink, maroon, and navy colors represent the existence of genes/replicon plasmids; white indicates the absence of gene/replicon plasmids. The figure was created using iTOL.

In silico analysis of multilocus sequence typing (MLST) using the Oxford scheme identified five sequence types (STs). The majority of isolates were attributed to ST773 (6/13) and ST235 (3/13), with two isolates classified as ST664, and sporadic occurrences of ST1822 and ST274 were noted (Fig. 1).

In silico serotyping analysis indicated that serotype O11 was predominant among the screened isolates (8/13; 61.5%), while serotypes O5, O7, O2, and O3 were each represented only once. (Fig. 1).

### Detection of antimicrobial resistance genes

Screening of antimicrobial resistance genes (ARGs) revealed the presence of *bla*_*NDM*_*, bla*_*PDC1*_*,* and *bla*_*PDC2*_ in 46.2% (6/13), 15.4% (2/13), and 69.2% (9/13) of screened isolates, respectively. Similarly, *bla*_*GEM*_ and *bla*_*SHV*_ were sporadically present in one isolate, whereas all the isolates carried *bla*_*OXA*_ type genes. Six isolates were found to carry aminoglycoside-modifying enzyme genes, such as *aadA11*, *ant(2’)-la* and *acc(6’)lb*. Additionally, 61.5% (8/13) of screened isolates contained the *rmtB* gene, which confers resistance to aminoglycosides.

All isolates were found to contain the genes *aph(3’)llb, armR*, *basS,* and *fosA*, which confer resistance to aminoglycosides, polymyxin, and fosfomycin, respectively. *Bla*_*OXA-488*_, *aph (3’)XV* and *aph(3’)lb* genes were found specifically in isolates belonging to ST235. On the other hand, ST773 exclusively carried *aadA10, rmtB, bla*_*PAO,*_* bla*_*OXA*-*395*_*,* and *qnrVC1,* with the exception of *qnrS1,* which was detected in two ST773 isolates. ST235 and ST773 were notable for exclusively harboring *sul1* and *bla*_*PDC2*_, respectively*.* On the other hand, ST664 possessed *bla*_*OXA-48*_, *bla*_*OXA-50*_ and *bla*_*PDC1*_. The *bla*_*VIM,*_*, bla*_*OXA-395*_, *qacE*, and *sul1* were found in ST1822 (Fig. 1). More resistance genes detected were detailed in Table S2.

### Mobilome associated with antibiotic resistance genes

**Figure 2:**
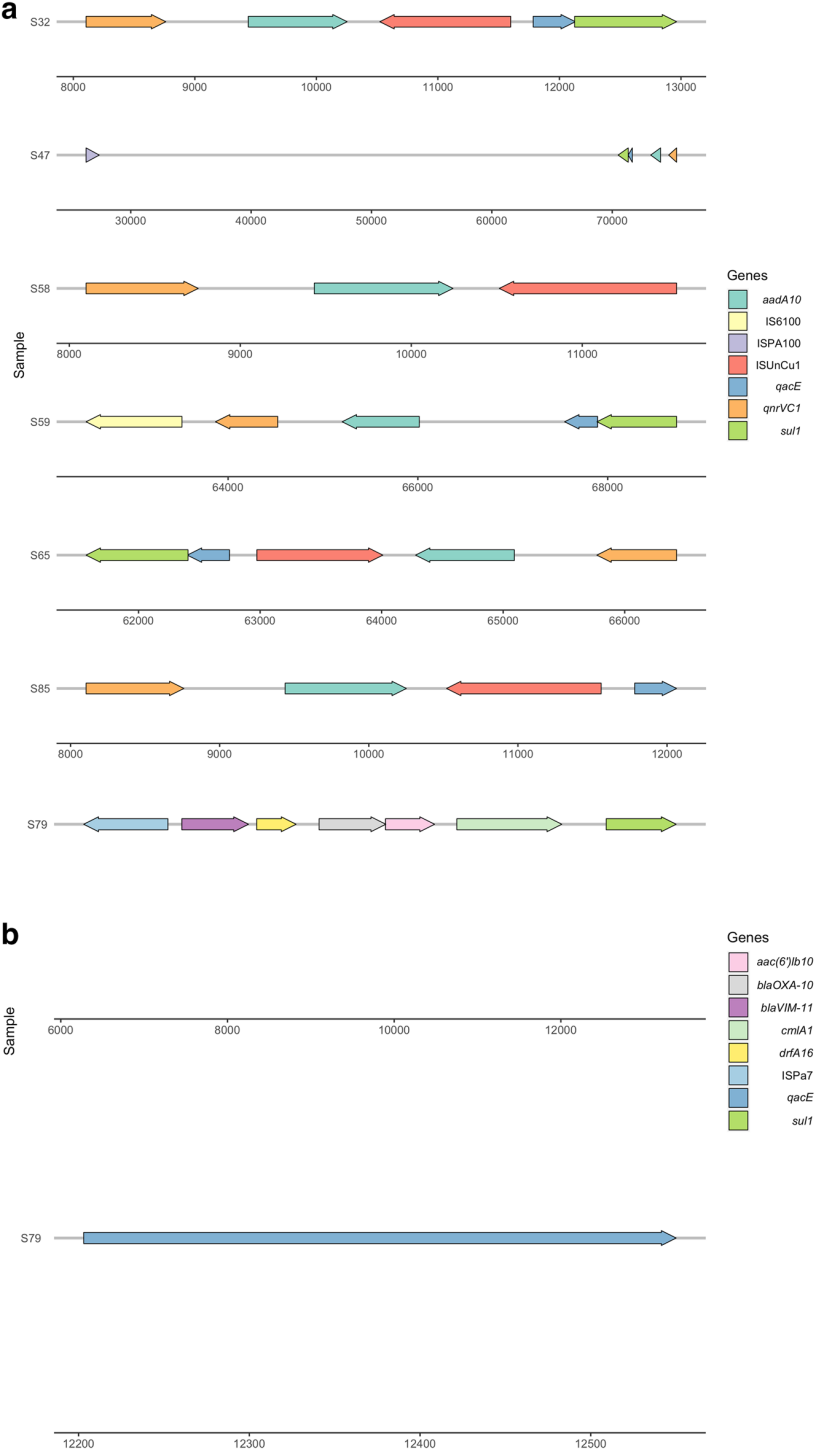
(**a**) Distribution of MGEs, including insertion sequences carrying different antimicrobial and antiseptic resistance genes were ST773. (**b**) Specific insertion sequences carrying ARGs found in ST1822. Both figures were created using ggplot2 and ggenes R packages.

Various mobile genetic elements (MGEs), including insertion sequences (IS), integrases, plasmids, and transposons, were detected across different STs. Specific insertion sequences (IS) carrying antibiotic resistance genes (ARGs) were identified. Notably, ST773 isolates possessed ISUnCu1, ISPA100 and IS6100, all of which carried acquired *qacEΔ, qnrVC1, sul1,* and *aadA10* genes (Fig. 2A). ST1822 isolate harbored ISPa7 which carried *sul1, bla*_*OXA-10*_* , aac(6’)-lb10, cmlA1, qacEΔ,* and *dfrA16* (Fig. 2B, Table S3). Remarkably, none of the isolates tested positive for the plasmid-mediated mobile resistance (*mcr*) gene.

### Distribution of plasmid replicons

Among *P. aeruginosa* isolates*,* multiple plasmids were identified including: IncF, IncQ, IncX, and Col. Interestingly, most of the isolates (8/13; 61.5%) harbored Col group plasmids, which included specific subtypes such as Col(pHAD28), Col440I, Col440II, ColpVC, ColRNAI, Col8282, and Col (KPHS6). The detected incompatibility (Inc) types were IncFII (Cf), IncX3, and IncQ, representing 15.4%, 15.4%, and 7.7% of the isolates, respectively (Fig. 1, Table S4).

### Single nucleotide polymorphisms in antimicrobial resistance determinants

Mutations in *ampR* gene, conferring resistance to aztreonam, along with the presence of *Pseudomonas*-derived cephalosporinase (*bla*_*PDC16/35*_) were prominently detected. Additionally, we identified single nucleotide polymorphisms (SNPs) that could potentially impact gene expression using the resistance gene identifier (RGI) (Table 1).

**Table 1:** Mutations associated with antibiotic resistance.

Gene	SNP/altered target	Antibiotic affected	Resistance mechanism
*gyrA*	P116A, T83I	Fluoroquinolone	Antibiotic target alteration
*gyrB*	I139R	Fluoroquinolone	Antibiotic target alteration
*parC*	S84L	Fluoroquinolone	Antibiotic target alteration
*parE*	V423F, I139R	Fluoroquinolone	Antibiotic target alteration
*mexR*	MexAB-OprM overexpression	Fluoroquinolone, aztreonam, carbapenem, cephalosporin	Antibiotic target alteration, Antibiotic efflux
*mexS*	MexEF-OprN overexpression	Fluoroquinolone	Antibiotic efflux
*nfxB*	MexCD-OprJ overexpression	Fluoroquinolone, aztreonam, carbapenem, cephalosporin	Antibiotic efflux
*mexZ*	MexXY-OprM overexpression	Aminoglycoside, fluoroquinolone, aztreonam, carbapenem, cephalosporin	Antibiotic efflux
*nalD*	MexAB-OprM overexpression	Fluoroquinolone, carbapenem, aminoglycoside, cephalosporin	Antibiotic efflux
*nalC*	MexAB-OprM overexpression	Fluoroquinolone, carbapenem, aminoglycoside, cephalosporin	Antibiotic efflux
*bla*_*PDC16/35*_	AmpC structural modification	Aztreonam, carbapenem, cephalosporin	Antibiotic inactivation
*ampR*	D135N, D135G	Aztreonam, carbapenem, cephalosporin	Antibiotic inactivation
*fusA*	V90I, H457Q	Aminoglycoside	Antibiotic target alteration

### Core genome MLST characterization

**Figure 3:**
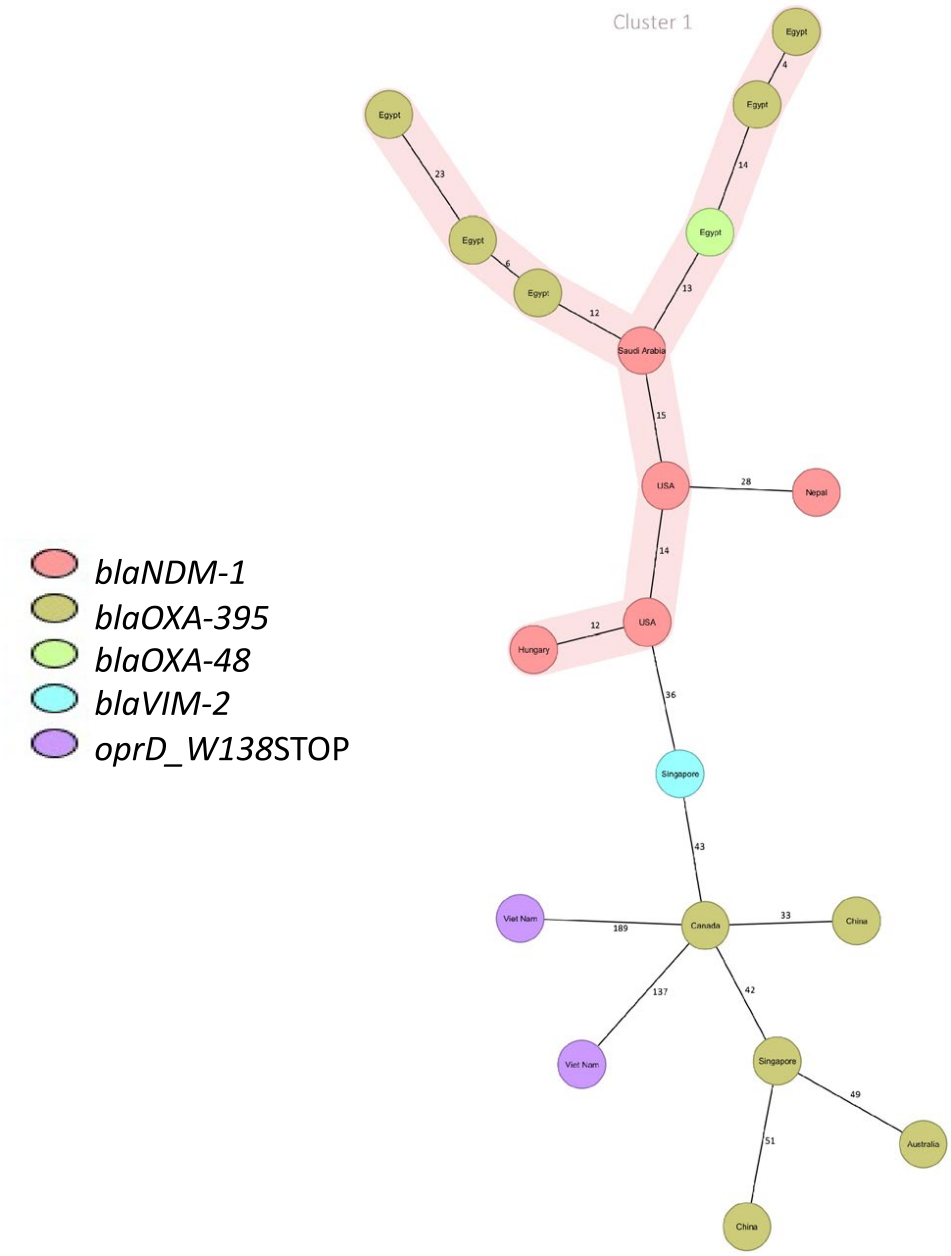
Minimum spanning tree (MST) of the 19 ST773 *P. aeruginosa* isolates (6 Egyptian and 13 worldwide isolates) collected from different geographical locations. Distances are based on the differences in the 3867 alleles in *P. aeruginosa* cgMLST, with a cluster distance threshold set at 25. Nodes are labeled by country of isolation, and colored based on the presence of carbapenem and beta-lactam resistance genes.

The relatedness of *P. aeruginosa* isolates assigned to ST773 and ST235 with globally reported isolates was investigated using the cgMLST scheme. This analysis involved a selected set of 13 publicly available *P. aeruginosa* isolates of ST773 from diverse geographical locations worldwide, and it included both cgMLST and phylogenetic reconstruction (Table S6). The analysis revealed a major cluster comprising our Egyptian isolates (Fig. 3), which clustered with isolates from Saudi Arabia. This genetic relevance can be attributed to geographical proximity. Additionally, the Egyptian isolates also clustered with isolates from the USA and Hungary, suggesting potential global expansion and adaptation (Fig. 3).

**Figure 4:**
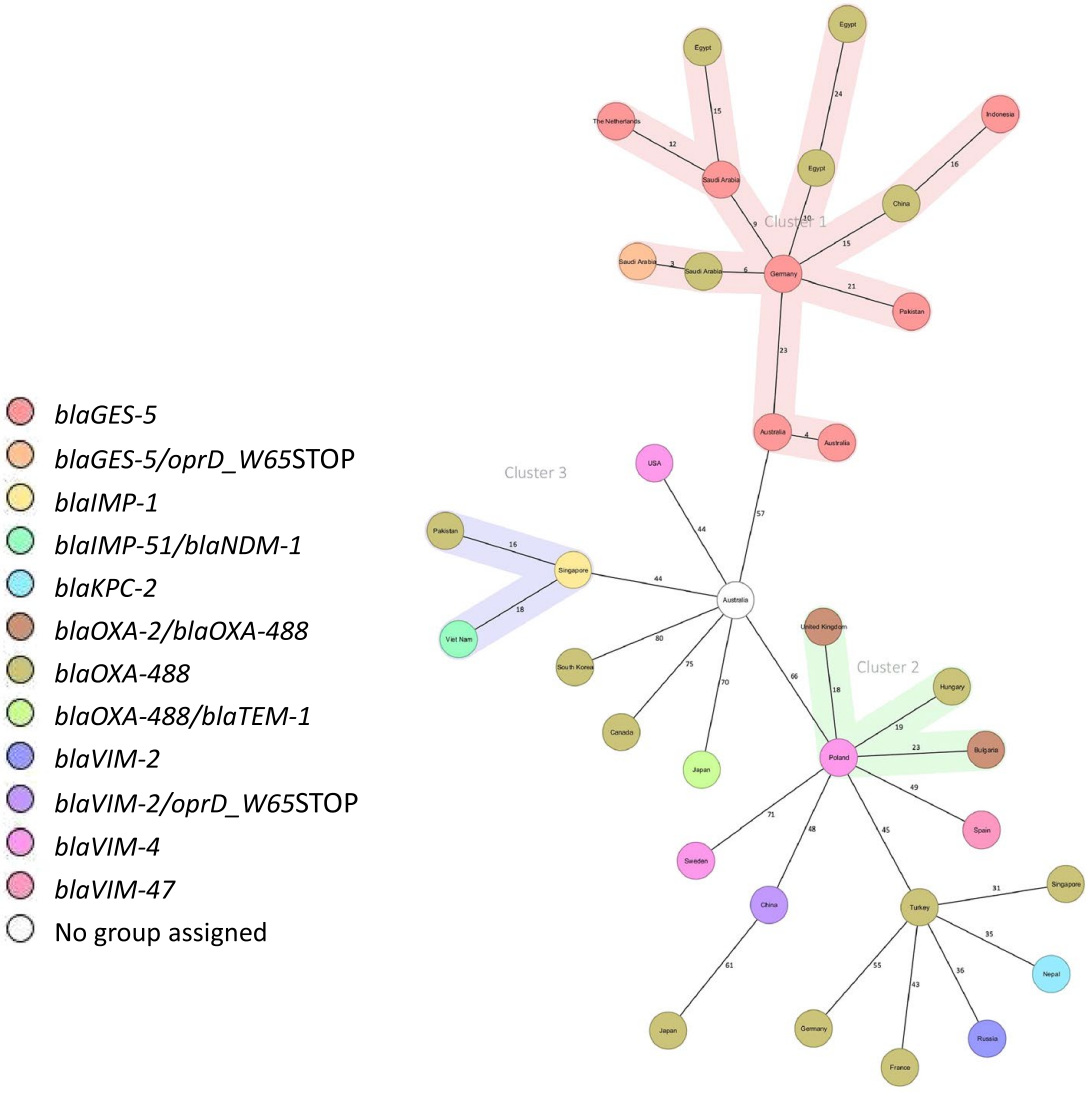
Minimum spanning tree (MST) illustrating the relatedness among 35 ST235.

Furthermore, cgMLST analysis was performed using a selected set of 32 publicly available *P. aeruginosa* isolates of ST235 reported worldwide (Table S7). The analysis identified 3 clonal clusters, Cluster 1 was the largest consisting of our Egyptian isolates grouping with isolates from Saudi Arabia, Netherlands, Australia, Pakistan, Germany, China, and Indonesia. The remaining isolates were considered unrelated to our isolates and assigned unique clonal clusters (Fig. 4).

*P. aeruginosa* isolates, comprising 3 Egyptian and 32 worldwide isolates from diverse geographic locations. Distances are based on the differences in the 3867 alleles in *P. aeruginosa* cgMLST, with a cluster distance threshold set at 25. Nodes are labeled by country of isolation, and colored based on the presence of carbapenem and beta-lactam resistance genes within the genome.

### Virulome analysis

**Figure 5:**
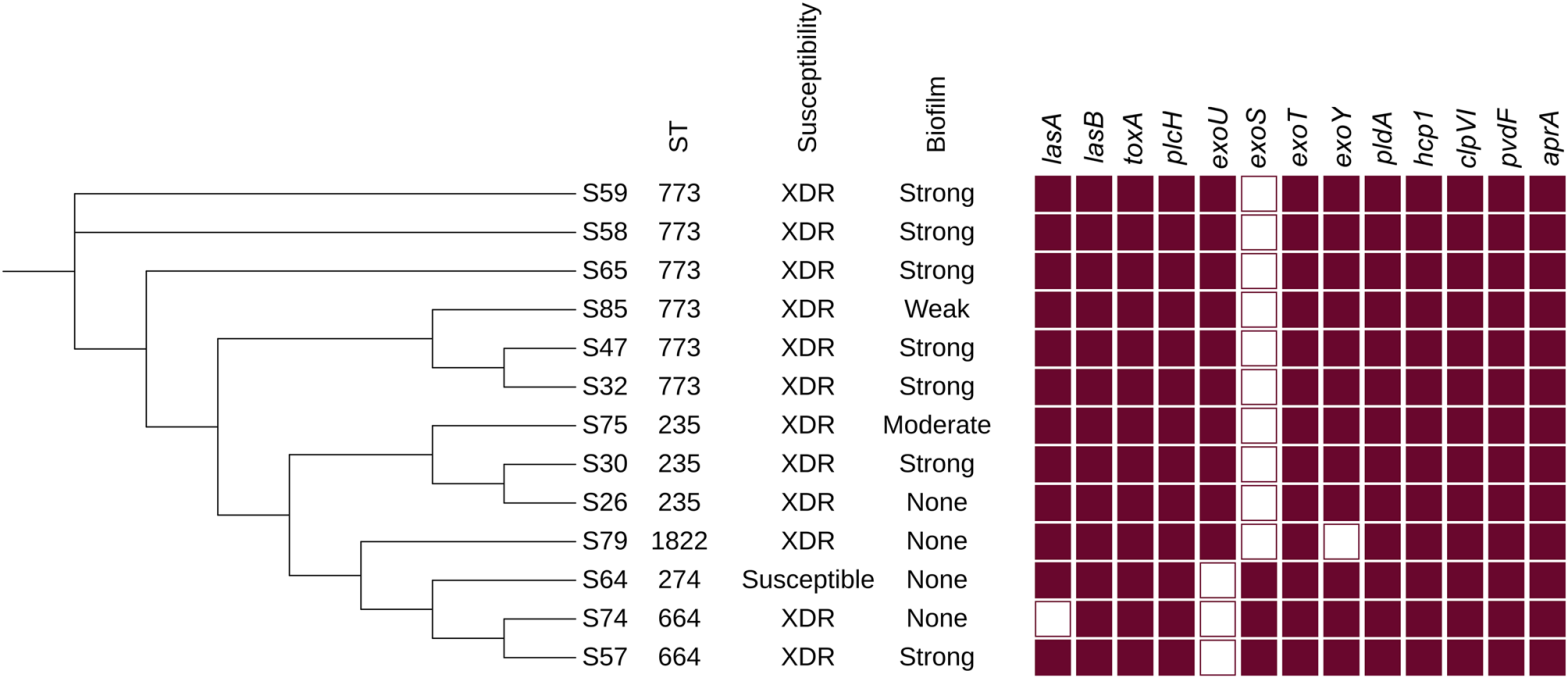
Presence of selected virulence genes and its correlation in the formation of biofilm. Maroon color showed the presence of these genes and white outlines the gene absence. Figure was created by iTOL.

Thirteen extensively drug-resistant (XDR) clinical isolates exhibited cytotoxic virulence patterns, with the detection of 87 virulence genes (Table S5) using the Virulence Factor Database (VFDB), of which only 15 were incorporated into Fig. 5. Among the genes encoding effector proteins that play a role in T3SS, *exoT* emerged as the most predominant virulence gene and was detected in all screened isolates. Among screened isolates, twelve isolates harbored *exoY*, ten carried *exoU*, and three contained *exoS*. Additionally, the phospholipase gene (*plcH*), exotoxin A gene (*toxA*), and elastase B gene (*lasB*) were detected in all *P. aeruginosa* isolates, except for the elastase A gene (*lasA*), which was found in only 12 isolates. The T6SS effector phospholipase (*pldA*), the effector gene in H1-T6SS (*hcp1*), and *ClpV1* were present in all isolates; however, *plcB* and *plcN* were absent in all isolates.

### Association between biofilm formation, clonal distribution, and AMR

We examined the correlation between biofilm formation (Table S8) and extensively drug-resistant (XDR) phenotypes among *P. aeruginosa* isolates (Fig. 5). More than half of the isolates (53.85%) exhibited strong biofilm production, particularly associated with ST235 and ST773.

## Discussion

In this study, we explored the population structure, virulence factors, and genomic alterations contributing to AMR, encompassing the acquisition of AMR genes through horizontal gene transfer and chromosomal mutations. Our findings highlight that the majority of our isolates exhibited XDR, aligning with a prior study documenting heightened resistance levels in *P. aeruginosa* strains originating from Egyptian clinical settings^23^. This poses significant challenges for infection control measures within the Egyptian health care settings and highlights the crucial need for enhanced infection prevention strategies, surveillance, and monitoring to mitigate the risk of spread of these pathogens.

Approximately 92.3% of our isolates demonstrated resistance to ticarcillin and ticarcillin-clavulanic acid, as well as to various antipseudomonal antibiotics including ciprofloxacin, gentamicin, cefepime, amikacin, ceftazidime, tobramycin, and piperacillin-tazobactam. Notably, the resistance patterns observed align with those reported in various observational studies on hospital-acquired *P. aeruginosa* in Egypt^23–25^. It underscores the challenge of treating infections caused by resistant *P. aeruginosa* isolates within the Egyptian healthcare settings. The concerning resistance rates against carbapenem antibiotics like imipenem and meropenem signify a potential obstacle to effective treatment, emphasizing the importance of genomic surveillance and antibiotic stewardship practices. All isolates were susceptible to colistin, the last resort for the treatment of carbapenem-resistant Gram-negative infections. This suggests that colistin remains an effective therapeutic choice for addressing *P. aeruginosa* infections in the Egyptian healthcare settings.

In this study, ST773 and ST235 emerged as the predominant clones, with serotype O11 being the most prevalent. Previous reports have linked ST235 clones and O11 serotype to multiple drug resistance^26^, global dissemination potential^26–30^ and Middle Eastern outbreaks^31–33^.

Using the proposed cgMLST scheme, we successfully linked our major reported clones (ST773 and ST235) to distant isolates reported worldwide. Although ST773 was not previously classified as a high-risk clone^34,35^, our study together with other recent studies^36,37^ suggests that ST773 is likely to emerge as a global clone. Furthermore, our findings indicate the local expansion of the ST773 lineage and the presence of diverse AMR determinants, enhancing its ability to propagate in epidemic settings. A major progression of the worldwide hyper virulent high-risk clone ST235 was documented in our study and several other studies^27,38^. This clone is playing a crucial role in the global prevalence of carbapenemase-producing isolates, and it has previously been associated with the acquisition of horizontally acquired resistance determinants across diverse geographical and environmental settings worldwide^39^.

Despite the limited number of isolates tested, the majority exhibited resistance to various antibiotics. Interestingly, each sequence type (ST) demonstrated a distinct profile of ARGs. For instance, ST773 isolates exclusively harbored *bla*_*OXA-395,*_* bla*_*PAO*_, *qnrVC1*, *rmtB,* and *aadA10;* while ST235 exclusively harbored *bla*_*OXA-488*_, *aph3’XV, aph3’lb* and *aph6’Id*, as well as *bla*_*PDC2*_ was only detected in both STs. This observation further supports the potential clonal dissemination of resistance determinants and propagation of resistance traits within a particular lineage that is induced, at least in part, by selective pressures of antibiotic use in the Egyptian healthcare settings.

Beside clonal dissemination of resistant clones, recent studies highlighted the role of MGEs, encompassing IS, transposons, integrases, phages, and integrative elements in the dissemination ARGs at the clinical settings^40,41^. In this study, different IS, including ISUnCu1, ISPA100, and IS6100 were detected in *P. aeruginosa* ST773 isolates, whereas the presence of ISPa7 was exclusively present in ST1822. ISUnCu1 was found within the *attC* sites of *aadA1*^40,42^; IS6100 was identified in gram-negative bacteria such as *Pseudomonas, Salmonella, Klebsiella, Acinetobacter* and *Enterobacter*, showing a variety of ARGs^41^; and in agreement with our results, ISPa7 has been reported on a upstream region from a *bla*_*VIM*_ gene^43^.

Our findings highlighted that a substantial number of isolates harbored Col plasmids from diverse subtypes, such as Col(pHAD28), Col440I, Col440II, ColpVC, ColRNAI, Col8282, and Col (KPHS6) were observed. These Col replicon subtypes have been previously associated with various carbapenemase genes, correlating with the observed high resistance to carbapenems in our study^44^. Consequently, these findings have significant implications for infection control strategies and antibiotic management. The high prevalence of resistance among our screened isolates, and the presence of plasmids and MGEs would facilitate the acquisition of new antimicrobial resistance determinants. Therefore, there is an urgent need for robust infection control measures and the implementation strict protocols to prevent the spread of resistant strains within healthcare settings.

Genetic determinants conferring resistance to various antibiotics, including β-lactams and carbapenems (*bla*_*TEM*_, *bla*_*CTX*-*M*_, *bla*_*NDM*_, *bla*_*IMP*_, *bla*_*PDC*_, *bla*_*VIM*_ and *bla*_*OXA48*_), fluoroquinolones (*gyrA/B*, and *parC/E* mutations), sulfonamides (*sul1*), trimethoprim (*dfrA*), tetracycline (*tet*A/B), phenicols (*cmlA*), and fosfomycin (*fosA*), were predominantly found in *Pseudomonas, Enterobacter* spp., *Klebsiella pneumoniae*, *Serratia marcescens*, *Escherichia coli*, *Salmonella enterica,* and *Acinetobacter baumannii*, carried by IncF-type, IncX_3/4_, ColRNAI, ColpVC plasmids^44–49^, similar to our findings. A recent investigation provided evidence supporting the widespread occurrence of fluoroquinolone resistance mediated by plasmids. Plasmid-based replicon typing was utilized to identify the plasmid incompatibility types of the transconjugants. Among the 12 transconjugants analyzed, 58.3% were classified as IncFII type plasmid replicons, 33.3% belonged to the IncA/C group, and 8.3% were categorized as IncFIC type. Additionally, our study identified the presence of IncFII and IncX3 harboring *bla*_*NDM*._ in two isolates, which aligns with previous studies^49–53^.

Previous studies have established a connection between fluoroquinolone resistance and DNA topoisomerase IV and gyrase SNPs^54–58^. Building on this foundation, our study offers additional insights into the impact of various chromosomal mutations on antimicrobial resistance, particularly in genes encoding DNA gyrase (gyrA-P116A and T83I, gyrB-I139R) and topoisomerase IV (parC-S84L, and parE-V423F and I139R). Furthermore, the presence of V90I and H457Q mutations in the *fusA* gene has been associated with aminoglycoside resistance. These mutations lead to altered elongation factor G (EF-G), potentially disrupting the normal translation process and reducing susceptibility to the inhibitory effects of aminoglycosides^57^. The causal relationship between *fusA* and aminoglycoside resistance was highlighted in a recent study employing site-directed mutagenesis^57–59^. Furthermore, we found an association that *ampR* with mutations in D135N and D135G, conferring resistant to aztreonam. Furthermore, among the isolates examined, at least one of the *bla*_*PDC16/35*_ genes were detected. Previous research has highlighted specific point mutations, such as D135N, that upregulate *ampC* gene expression, thereby enhancing resistance to β-lactam antibiotics^56^.

*P. aeruginosa* possesses sophisticated secretion systems that facilitate the delivery of virulence factors, including toxins, elastases, lipases, and proteases into hots cells^8^. Consequently, our virulome analysis revealed the presence of virulence genes, shedding light on its pathogenicity and antibiotic resistance profiles. *P. aeruginosa* utilizes the T3SS to deliver its effector toxins (exoT, exoY, exoS, and exoU) into the host cells. This mechanism facilitates colonization and aids evasion from the immune system^60^. Interestingly, *exoT* was the most prevalent gene, along with *exoY* (except in S79). Additionally, *exoS* and *exoU* were found to be mutually exclusive.

Furthermore, a recent study revealed that *P. aeruginosa* utilizes H1-, H2-, and H3-T6SS to transport its toxins into both eukaryotic and prokaryotic cells via effectors pldA, hcp1, and ClpV1^61^*.* Consistent with these findings, we detected these encoding genes in our isolates. Additionally, the T2SS of *P. aeruginosa* is crucial for the release of different effector proteins, which play a significant role in damaging host cells and contributing to the pathogenicity of *P. aeruginosa*^62^. In this study, we observed the presence of *toxA*, *lasB*, and *plcH* in all screened isolates, while *lasA* was found in 12 isolates.

In conclusion, our pilot study has provided several significant insights into the differential clonal distribution of resistant *P. aeruginosa* isolates. It highlights the pivotal role of horizontal gene transfer and MGEs in bacterial adaptation, particularly in response to the selective pressure induced by antibiotic overuse in clinical settings, especially in low- or middle-income countries. These findings collectively underscore the urgent need for enhanced surveillance and strategic interventions to curb the spread of XDR *P. aeruginosa* in healthcare settings, given the complexity and adaptability of these bacterial strains. Further research and ongoing monitoring are imperative for a comprehensive understanding of the mechanisms driving antimicrobial resistance and the development of effective control strategies.

## Methods

### Bacterial isolation and identification

Thirteen non-duplicate historical *P. aeruginosa* isolates were collected from one microbiology laboratory serving as a diagnostic facility for multiple hospitals in Alexandria, Egypt, between August 2020 and March 2021, as previously mentioned^63^. Patient names remained anonymous.

These isolates were obtained from aspirate, urine, pleural fluid, upper limb post-burn, neck wound, diabetic foot, and vertebral surgical lesion specimens. Species identification was confirmed using the VITEK 2 Compact GN ID card (bioMérieux, Marcy-l’Étoile, France). All isolates were preserved at − 80 °C in brain heart infusion broth (BHB) containing 10% glycerol (HiMedia, India) until further analysis.

### Antimicrobial susceptibility testing (AST)

Minimum inhibitory concentration (MIC) method for screened isolates were obtained using VITEK 2 Compact GN ID card (bioMérieux, Marcy-l’Étoile, France). The antibiotic susceptibility panel contained fourteen antibiotics, including ticarcillin (TIC, ≥ 128 µg/mL), ticarcillin-clavulanic acid (TIM, ≥ 128 µg/mL), piperacillin (PIP, ≥ 128 µg/mL), piperacillin-tazobactam (TZP, ≥ 128 µg/mL), ceftazidime (CAZ, ≥ 64 µg/mL), cefepime (FEP, ≥ 64 µg/mL), amikacin (AMI, ≥ 64 µg/mL), gentamicin (GEN, ≥ 16 µg/mL), tobramycin (TOB, ≥ 16 µg/mL), ciprofloxacin (CIP, ≥ 4 µg/mL), aztreonam (ATM, ≥ 64 µg/mL), imipenem (IPM, ≥ 16 µg/mL), meropenem (MER, ≥ 16 µg/mL), and colistin (COL, ≤ 0.5 µg/mL).

Antibiotic resistance patterns are categorized into three main classes: MDR (Multi-Drug Resistance), characterized by resistance to at least one drug in three antibiotic classes; XDR, indicating resistance to most antibiotics except one or two classes; and PDR (Pan-Drug Resistance), indicating resistance to all available antibiotic agents^64^.

Results were interpreted following the guidelines outlined by the Clinical and Laboratory Standards Institute (CLSI, 2023). The tested antipseudomonal antibiotics included cephalosporins (ceftazidime and cefepime), penicillin (piperacillin), penicillin combined with β-lactamase inhibitors (ticarcillin, ticarcillin-clavulanic acid, and piperacillin-tazobactam), monobactams (aztreonam), carbapenems (imipenem and meropenem), aminoglycosides (amikacin, tobramycin, and gentamicin), fluoroquinolones (ciprofloxacin), and lipopeptide (colistin)^65^. The modified carbapenem inactivation method was performed following the CLSI 2023 guidelines to confirm carbapenemase production among *P. aeruginosa* isolates. *Escherichia coli* ATCC 25,922 was included as a quality control for the assay^66^.

### In vitro biofilm screening

Biofilm screening among tested isolates was conducted as previously described^67,68^ with some modifications. Briefly, 20 µl of each bacterial isolate was added to 180 µl of fresh brain heart infusion broth (BHB) in a 96-well plate and incubated for 24 h at 37 °C. Subsequently, the wells were washed with 1X phosphate-buffered saline (PBS; pH 7.2) to remove planktonic cells and then dried in a static incubator. Next, 200 µl of 0.1% crystal violet (Sigma-Aldrich, USA) was added to each well and left for 30 min at room temperature.

Briefly, 20 µl of each bacterial isolate was added into 180 µl fresh BHB in a 96 well plate and incubated for 24 h at 37 °C. Afterwards, 1X phosphate buffer saline (ThermoFisher Scientific, US) (PBS; PH 7.2) was used to wash the wells from planktonic cells, dried in the static incubator. Then, 200 µl of 0.1% crystal violet (Sigma-Aldrich, USA) was added to each well and left for 30 min at room temperature. Subsequently, each well was gently rinsed three times with sterilized 1X PBS and left overnight to dry. Lysis solution (10% SDS, 99% Ethanol and 1X PBS) was added to each well and laid on the plate shaker for an hour. The optical density of each well was measured (λmax = 595 nm) using the microplate reader (FLUOstar^®^ Omega, BMG LABTECH, Germany). The experiments were repeated in triplicates and the biofilm formation index was calculated. The low cut-off (ODc) was calculated as three standard deviation (3xSD) above the mean of OD of negative control. Isolates were categorized into non-biofilm producer (OD ≤ ODc), weak biofilm producer (Odc < OD ≤ 2xODc), moderate biofilm producer (2xODc < OD ≤ 4xODc), and strong biofilm producer (4xODc < OD)^66^. *E. coli* DH5-alpha and *P. aeruginosa* (ATCC10145 and ATCC9027) were used as negative and positive controls, respectively.

### Whole genome sequencing, assembly, and annotation

The genomic DNA of the thirteen selected *P. aeruginosa* isolates was extracted using the QIAamp DNA Mini Kit (Qiagen, UK) following the manufacturer's protocol, as previously described^63^. Briefly, DNA quality and concentration were assessed using a Qubit 3.0 Fluorometer (Thermo Fisher Scientific, USA). To prepare the libraries, one μg of genomic DNA was used, following the manufacturer’s instructions. The NEXTflex Rapid XP DNA-Seq library Preparation Kit was used for library preparation (PerkinElmer, USA). Whole-genome sequencing was performed on the Illumina NextSeq 550 platform (Illumina Inc., San Diego, CA) using the NextSeq 500/550 mid output kit v2.5 (300 cycles) paired-end kit. Then, fastp was used to remove the adaptors and low-quality reads^69^. The de novo assembly of *P. aeruginosa* genomes was performed using Unicycler (version 0.4.8)^70^, and assembly metrics were generated using QUAST^71^.

### Multilocus sequence typing, serotyping and integrative mobile genetic elements analysis

The PubMLST database (https://pubmlst.org/)^72^ was used to assign the sequence type of each bacterial isolate. This involved uploading contigs and assigning the alleles of the seven housekeeping genes (*acsA, aroE, guaA, mutL, nuoD, ppsA,* and *trpE*) following the Oxford scheme. Sequences were submitted to PubMLST database (IDs: 8978, 8980, 8983, 8977, 8981, 8982, 8985, 8986, 8987, 8988, 8979, and 8984). Detection of *P. aeruginosa* serotype (PAst) was performed using Center for Genomic Epidemiology (CGE) (https://cge.food.dtu.dk/services/PAst/)^73^.

Mobile genetic elements (MGEs) and their genetic context with antibiotic resistance genes were determined using Mobile Element Finder (https://cge.food.dtu.dk/services/MobileElementFinder/)^74^ and EggNOG-Mapper (https://github.com/eggnogdb/eggnog-mapper)^75^ while replicon typing was detected using ABRicate (https://github.com/tseemann/abricate) and PlasmidFinder^76^. Insertion sequences were identified using ISFinder tool (https://www-is.biotoul.fr/index.php)^77^.

### Annotation and pangenome analysis

Contigs were annotated using Prokka^78^, and the GFF3 output files from Prokka were utilized for the pangenome analysis of the isolates. The Roary software^79^ was employed to derive pangenome statistics for core, shell, and cloud genes.

### In silico identification of resistome and virulome

Genome resistomes were analyzed using ABRicate (https://github.com/tseemann/abricate) with ResFinder and Comprehensive Antibiotic Resistance Database (CARD) databases^80,81^. Removal of redundant genes from both databases was performed. Resistance genes were categorized based on the antibiotic classes. Additionally, chromosomal mutations and SNPs for AMR in each clinical isolate were identified by extracting the sequences of the selected resistance genes and running CARD resistance gene identifier (RGI)^81^. The virulome profile of the isolates was annotated based on VFDB in ABRicate. Virulence genes were organized according to virulence factors and phenotypes.

### Phylogenetic tree construction

cgMLST was performed by fast-GeP. Core genome polymorphic genes (genes with at least one nucleotide difference among all genomes) were selected and concatenated by the ruby script concat_cgMLST_genes.rb (https://github.com/JoseCoboDiaz/concat_cgMLST_genes). The concatenated gene-by-gene fasta file was used for alignment and phylogenetic tree building using MAFFT version 7 (using default parameters for alignment and the Neighbor-Joining method, the Jukes-Cantor substitution model and 1000 bootstrap resampling for the construction of the phylogenetic tree)^82^. The cgMLST tree was visualized using iTOL (https://itol.embl.de/)^83^.

### Core genome MLST global comparison of predominant clones

The cgMLST comparison of ST773 and ST235 with globally reported isolates was performed using a well-defined scheme available in Ridom SeqSphere + v.8.3.5 software (Ridom GmbH, Münster, Germany), according to the *P. aeruginosa* sensu lato cgMLST’ version 1.0 scheme https://www.cgmlst.org/ncs/schema/schema/Paeruginosa783/ which included 3867 genes of the *P. aeruginosa* core genome (cgMLST). Seqsphere + tool mapped the reads against the reference genome using BWA v 0.6.2 software (parameters setting: minimum coverage of five and Phred value > 30) and defined the cgMLST gene alleles. A combination of all these alleles in each isolate formed an allelic profile that was utilized to create a minimum spanning tree (MST) using Ridom SeqSphere + with the ‘pairwise ignore missing values; % column difference’ parameter. A threshold was set at ≤ 15 allelic differences paired with a cluster alert quality threshold of at least 85% good cgMLST targets to define the clusters.

### Supplementary Information

Supplementary Information.

## Data Availability

The raw sequencing data of this study were submitted to the National Center for Biotechnology Information (NCBI) Bioproject database. The Bioproject accession number assigned to these data is PRJNA906142. The SRA accession numbers of isolates analyzed in this study were provided in Supplementary Table S1.
